# BastionHub: a universal platform for integrating and analyzing substrates secreted by Gram-negative bacteria

**DOI:** 10.1093/nar/gkaa899

**Published:** 2020-10-21

**Authors:** Jiawei Wang, Jiahui Li, Yi Hou, Wei Dai, Ruopeng Xie, Tatiana T Marquez-Lago, André Leier, Tieli Zhou, Von Torres, Iain Hay, Christopher Stubenrauch, Yanju Zhang, Jiangning Song, Trevor Lithgow

**Affiliations:** Infection and Immunity Program, Biomedicine Discovery Institute and Department of Microbiology, Monash University, VIC 3800, Australia; Infection and Immunity Program, Biomedicine Discovery Institute and Department of Microbiology, Monash University, VIC 3800, Australia; Department of Clinical Laboratory, The First Affiliated Hospital of Wenzhou Medical University, Wenzhou, Zhejiang Province, China; School of Computer Science and Information Security, Guilin University of Electronic Technology, Guilin 541004, China; School of Computer Science and Information Security, Guilin University of Electronic Technology, Guilin 541004, China; Infection and Immunity Program, Biomedicine Discovery Institute and Department of Microbiology, Monash University, VIC 3800, Australia; School of Computer Science and Information Security, Guilin University of Electronic Technology, Guilin 541004, China; School of Computer Science and Information Security, Guilin University of Electronic Technology, Guilin 541004, China; Department of Genetics, School of Medicine, University of Alabama at Birmingham, AL, USA; Department of Cell, Developmental and Integrative Biology, School of Medicine, University of Alabama at Birmingham, AL, USA; Department of Genetics, School of Medicine, University of Alabama at Birmingham, AL, USA; Department of Cell, Developmental and Integrative Biology, School of Medicine, University of Alabama at Birmingham, AL, USA; Department of Clinical Laboratory, The First Affiliated Hospital of Wenzhou Medical University, Wenzhou, Zhejiang Province, China; Infection and Immunity Program, Biomedicine Discovery Institute and Department of Microbiology, Monash University, VIC 3800, Australia; School of Biological Sciences, The University of Auckland, Auckland 1010, New Zealand; Infection and Immunity Program, Biomedicine Discovery Institute and Department of Microbiology, Monash University, VIC 3800, Australia; School of Computer Science and Information Security, Guilin University of Electronic Technology, Guilin 541004, China; Monash Centre for Data Science, Faculty of Information Technology, Monash University, VIC 3800, Australia; Infection and Immunity Program, Biomedicine Discovery Institute and Department of Biochemistry and Molecular Biology, Monash University, VIC 3800, Australia; ARC Centre of Excellence in Advanced Molecular Imaging, Monash University, VIC 3800, Australia; Infection and Immunity Program, Biomedicine Discovery Institute and Department of Microbiology, Monash University, VIC 3800, Australia

## Abstract

Gram-negative bacteria utilize secretion systems to export substrates into their surrounding environment or directly into neighboring cells. These substrates are proteins that function to promote bacterial survival: by facilitating nutrient collection, disabling competitor species or, for pathogens, to disable host defenses. Following a rapid development of computational techniques, a growing number of substrates have been discovered and subsequently validated by wet lab experiments. To date, several online databases have been developed to catalogue these substrates but they have limited user options for in-depth analysis, and typically focus on a single type of secreted substrate. We therefore developed a universal platform, BastionHub, that incorporates extensive functional modules to facilitate substrate analysis and integrates the five major Gram-negative secreted substrate types (i.e. from types I–IV and VI secretion systems). To our knowledge, BastionHub is not only the most comprehensive online database available, it is also the first to incorporate substrates secreted by type I or type II secretion systems. By providing the most up-to-date details of secreted substrates and state-of-the-art prediction and visualized relationship analysis tools, BastionHub will be an important platform that can assist biologists in uncovering novel substrates and formulating new hypotheses. BastionHub is freely available at http://bastionhub.erc.monash.edu/.

## Graphical Abstract

**Graphical Abstract:**
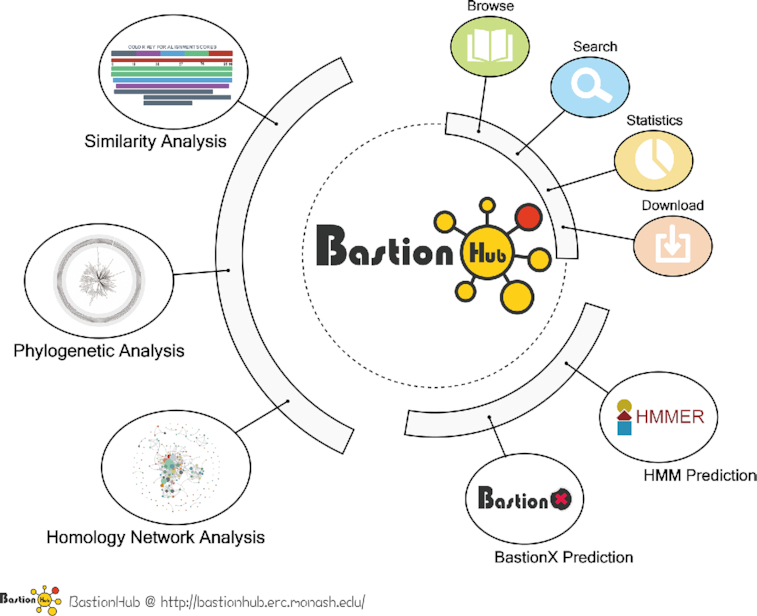
BastionHub: a universal platform for integrating and analyzing substrates secreted by Gram-negative bacteria.

## INTRODUCTION

Secretion systems are one of the key ‘weapons’ used by bacteria to unleash a repertoire of virulence factors into eukaryotic host cells or into neighboring bacterial cells to disrupt their normal cellular processes (1). To date, nine secretion system types have been discovered (T1SS to T9SS) (2–6), but only six of these are predominantly involved in the release of a secreted substrate into the extracellular environment. Gram-negative bacteria typically use T1SS-T4SS and T6SS to secrete substrates into the surrounding environment (T1SS-T2SS) or into other cells (T3SS, T4SS and T6SS) (2), whereas this purpose is fulfilled by the T7SS in select Gram-positive bacteria (including *Mycobacterium* spp.) (3). The three remaining classes of substrates, ‘secreted’ by T5SS, T8SS or T9SS, are not always released from the cell. In T5SS, there are five substrate subclasses (5a–5e) that either remain attached to the bacterium and are involved in attachment to other cells or surfaces (subclasses 5c and 5e, and some members of 5a) or are alternatively released into the extracellular medium (subclasses 5b and 5d, and some members of 5a) (4). T8SS secrete curli fibers that aggregate to form a complex extracellular matrix involved in surface adhesion and biofilm formation (5), whereas T9SS substrates appear to be restricted to the Bacteroidetes phylum where they either remain attached to the cell surface to facilitate gliding motility or are secreted into the extracellular medium (6).

Proteins secreted by secretion systems are globally known as ‘substrates’, but if the substrate mimics a host-cell function, like those from T3SS, T4SS and T6SS, it is instead referred to as an ‘effector’. Despite this distinction, ‘substrate’ and ‘effector’ have become largely interchangeable terms in the bacterial secretion system field. In this work, while we have tried to uphold this distinction, we do incorporate the term ‘effector’ when used to abbreviate the substrates of T1SS–T6SS (T1SE–T6SE) to be consistent with previous literature and databases. Every substrate/effector that belongs to a secretion system and, very often the structural components of secretion systems, are encoded within an operon: a series of genes set in a chromosomal or plasmid locus so that positional information of the gene context can be useful in identifying the components of the secretion system. Furthermore, some of the substrate proteins secreted by these secretion systems are encoded from genes located in this same gene context. An example is the substrate of the T2SS, PulA, which is encoded by a gene in the operon for the structural components of the T2SS in *Klebsiella* (7). Furthermore, other loci or ‘genomic islands’ sometimes encode several substrates for controlled expression (8) and, again, the positional information of a gene encoding a candidate substrate/effector can therefore be an additional clue in the case to investigate a candidate with wet-lab experiments.

Considering that secreted substrates vary in sequence, structure, mechanism and function, it is not surprising that there is no universal platform that integrates the various types of secreted effectors (9,10). Among those available, T3SEdb (11), T3DB (12) and BEAN 2.0 (13) catalogue different sets of validated T3SEs, whereas SecReT4 (14) and SecReT6 (15) focus on validated T4SEs and T6SEs, respectively. SecretEPDB (16) integrates a more comprehensive list of validated T3SEs, T4SEs and T6SEs, while EffectiveDB (17,18) contains the largest list of predicted T3SEs, T4SEs and T6SEs (although these predictions overlap with experimentally validated effectors). The majority of these toolkits allow users to browse an annotated list of validated proteins, but some also allow users to predict novel secreted substrates. While these platforms have been used to varying success by biologists, they are usually restricted to a single type of secreted substrate, and rarely include investigative capabilities for in-depth substrate analysis and visualization.

Here, we present BastionHub, a universal platform to integrate and analyze the five major types of substrates secreted by Gram-negative bacteria. By manually mining current literature and curated datasets, we collected detailed annotations for T1SE–T4SE and T6SE. These details were fully incorporated into BastionHub where users can browse, search, download, and view informative statistics and detailed information to facilitate their investigation into secreted substrates. We then developed and integrated two types of prediction tools: a hidden Markov model (HMM) based predictor to identify homologous substrates and the machine-learning based predictor, BastionX, that can alternatively be used to identify distantly related (and sometimes unrelated) substrates. Finally, we designed and implemented three data visualization tools to facilitate relationship analyses: a sequence similarity analysis tool, a phylogenetic analysis tool and a homology network analysis tool, which are fully interactive and designed to facilitate substrate investigation and analysis. By comprehensively integrating the various investigation and functional modules into a pipeline, BastionHub can provide an all-in-one service for users to analyze known substrates, predict new substrates and easily visualize their functional relationships.

## MATERIALS AND METHODS

**Figure 1.**
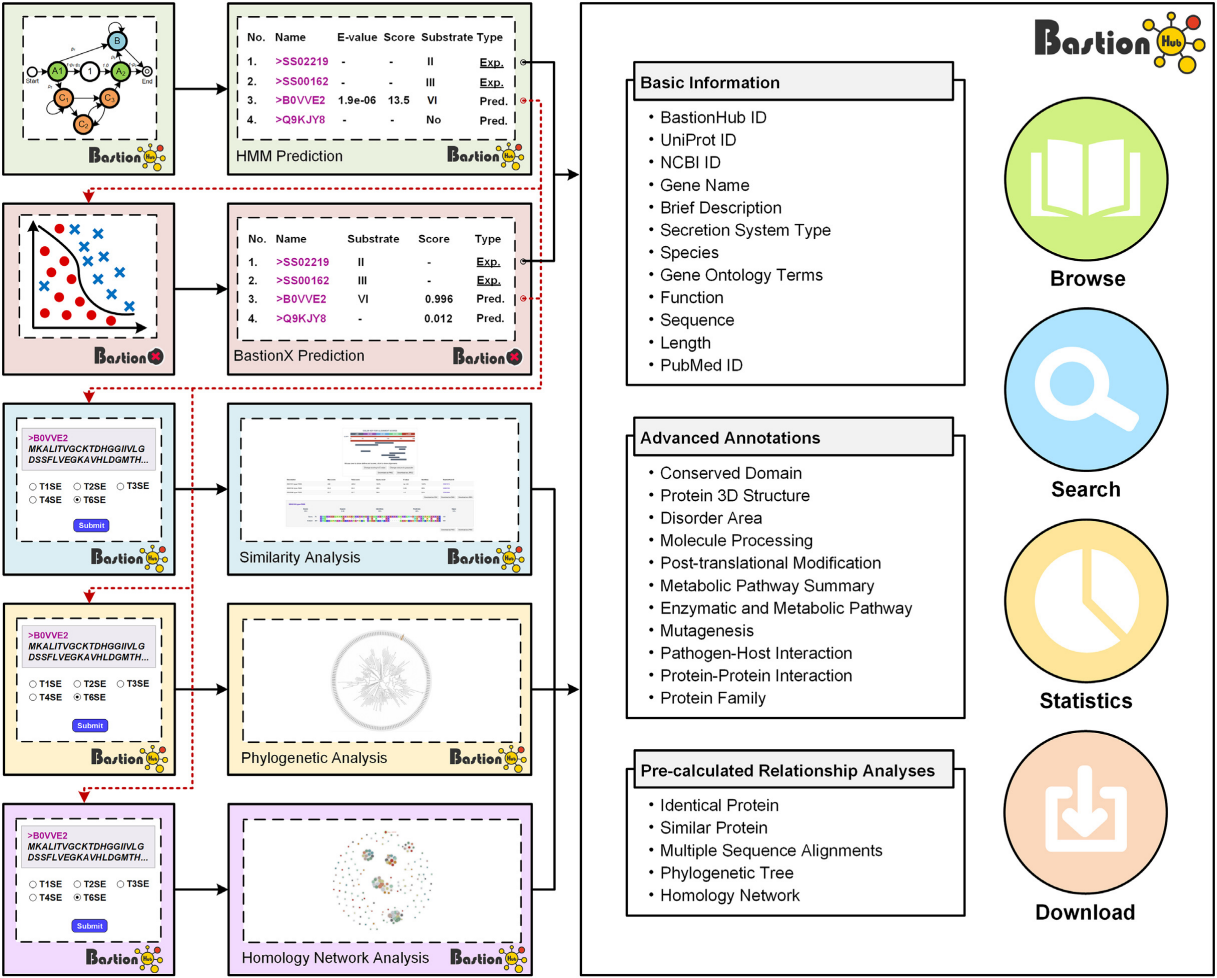
General framework of BastionHub illustrating the interconnecting modules that provide standard substrate investigation modules (right panel) and advanced functional modules (left panels). Solid lines indicate procedures within each functional model that operate as an independent toolkit, while dotted lines highlight interactions between different functional modules as interconnecting pipelines.

The overall BastionHub workflow consists of three steps: data collection and curation, data annotation, and website design and implementation (Figure 1).

### Data collection and curation

**Figure 2.**
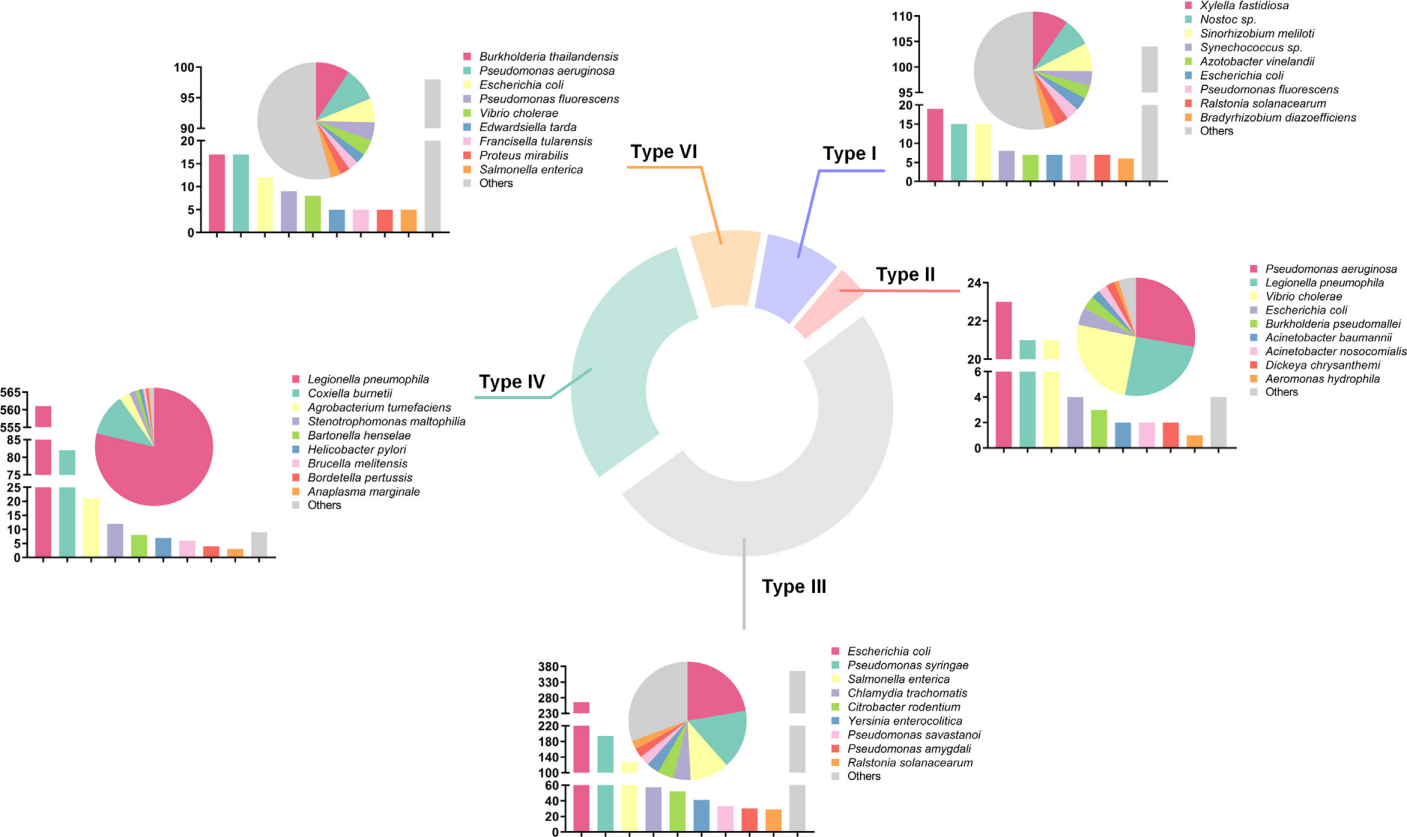
Distribution of the 2366 validated secreted substrates catalogued in BastionHub. The doughnut chart illustrates the proportion of each type of secreted substrate. Each subgraph shows the species distribution for that secretion type as a proportion (pie chart) or by total numbers (bar chart).

We systematically reviewed existing literature about T1SEs or T2SEs, which was made particularly difficult because there are no uniform names for these secreted substrates. We identified >5000 unique references and, after examining each text, we obtained 195 T1SEs across 63 species and 83 T2SEs across 13 species (Figure 2).

From available web resources listing T3SEs, T4SEs and T6SEs, we extracted details for each substrate to obtain a preliminary dataset. For any entry not annotated with both UniProt ID (19) and NCBI Protein ID (20), we used BLAST to identify an identical sequence from the same species to obtain the missing ID code if available. After manually inspecting each individual annotation, we removed obvious errors (e.g. those annotated as ‘membrane’ proteins or ‘secretion chaperone’ proteins). We then annotated the remaining entries with their associated PubMed reference ID where available. Similar to that for T1SE and T2SE, we conducted an exhaustive literature search and retrieved the most recent experimentally validated substrates, including substrates that had previously been overlooked. Accordingly, we obtained 1194 T3SEs across 72 species, 713 T4SEs across 15 species and 181 T6SEs across 66 species (Figure 2). Altogether, we obtained 2366 substrates secreted by the five secretion systems across 171 species (Figure 2). These substrates were then incorporated into BastionHub, and their annotations can be found on their dedicated *Detailed information* page.

### Data annotation

Beyond keeping basic information that at least one other database also includes (e.g. SecretEPDB), we incorporated additional experimental data for each substrate if available. Some of those annotations were assembled by the UniProt database (19) from different sources. Conserved domain data was collected from the Pfam database (21) and visualized by the IBS tool (22). Tertiary structures were collected from the protein data bank (PDB) (23). Enzymatic and metabolic pathways were collected from the BioCyc (24) and BRENDA (25) databases. Pathogen–host interaction data was collected from the PHI-base database (26). Putative protein-protein interactions were collected from the STRING (27), DIP (28), IntAct (29) and MINT (30) databases. The remaining data was collected directly from UniProt database, including potential molecule processing and post-translational modification information, metabolic pathway summaries, and details about mutagenesis studies within each substrate. Finally, we annotated each substrate with their references (where available) from PubMed (20).

For each substrate, we also included predicted annotations and pre-calculated relationship analyses against known substrates. The natively disordered area was predicted by the IUPred2A server and visualized using ECharts (https://echarts.apache.org/). The BLAST tool (version 2.8.1+) (31) was used to search against known substrates to calculate sequence similarities, which was visualized by BlasterJS (32). Homologous sequences were then used to generate a multiple sequence alignment file and then visualized with the R library msa (33). The MAFFT tool (version v7.271) (34) was used to generate the multiple sequence alignment results against known substrates, from which the phylogenetic tree structure was inferred by FastTree (version 2.1.8) (35) and then visualized by jsPhyloSVG (36). All-against-all BLAST (version blast-2.2.26) (37) was used to compare the query protein with known substrates to generate a sequence homology network, which was then visualized by ECharts. Pairwise sequence alignments between linked nodes in the network were generated using the EMBOSS Stretcher web service (38).

### Website design and implementation

BastionHub uses a data-oriented architecture with multiple functional modules, including standard investigation modules and advanced functional modules.

BastionHub was implemented using the JAVA (https://www.java.com/) server development suite, including the business logic layer controlled by Struts 2 (https://struts.apache.org/) and the model layer supported by Hibernate (https://hibernate.org/). This was accompanied with the view layer implemented by JSP, CSS, the JavaScript library jQuery (https://jquery.com/), the front end framework Bootstrap (https://bootstrapdocs.com/) and their libraries and packages. The MySQL database (https://www.mysql.com/) was used to store all substrates and their annotations.

The advanced functional modules were developed using additional techniques. For fast homologue identification, we constructed a set of HMM based models using HMMER (39) to predict potential type I, II, III, IV and VI substrates. We further integrated a suite of algorithms BastionX (http://bastionx.erc.monash.edu/) to enable more accurate substrate prediction. BastionX takes advantages of existing single type substrate predictors (40–43), and further develops T1SE and T2SE predictors to comprehensively predict all five types of secreted substrates from Gram-negative bacteria. The three relationship analysis tools were implemented using multiple programs with different steps, which have been detailed in the section *Data annotation*. Those time-consuming steps in each of the advanced functional modules were streamed by Perl CGI (https://metacpan.org/pod/CGI) based threads, and detached from the web interface using a queueing system implemented by the Gearman framework (http://gearman.org/).

## RESULTS

The BastionHub platform includes *Home*, *Substrate investigation*, *Prediction*, *Relationshipanalysis*, *Help* and *Contact* modules. All functional modules can operate independently or collectively within the BastionHub pipelines (Figure 1), which are detailed within the *Help* module and described as follows.

### Basic investigation modules

**Figure 3.**
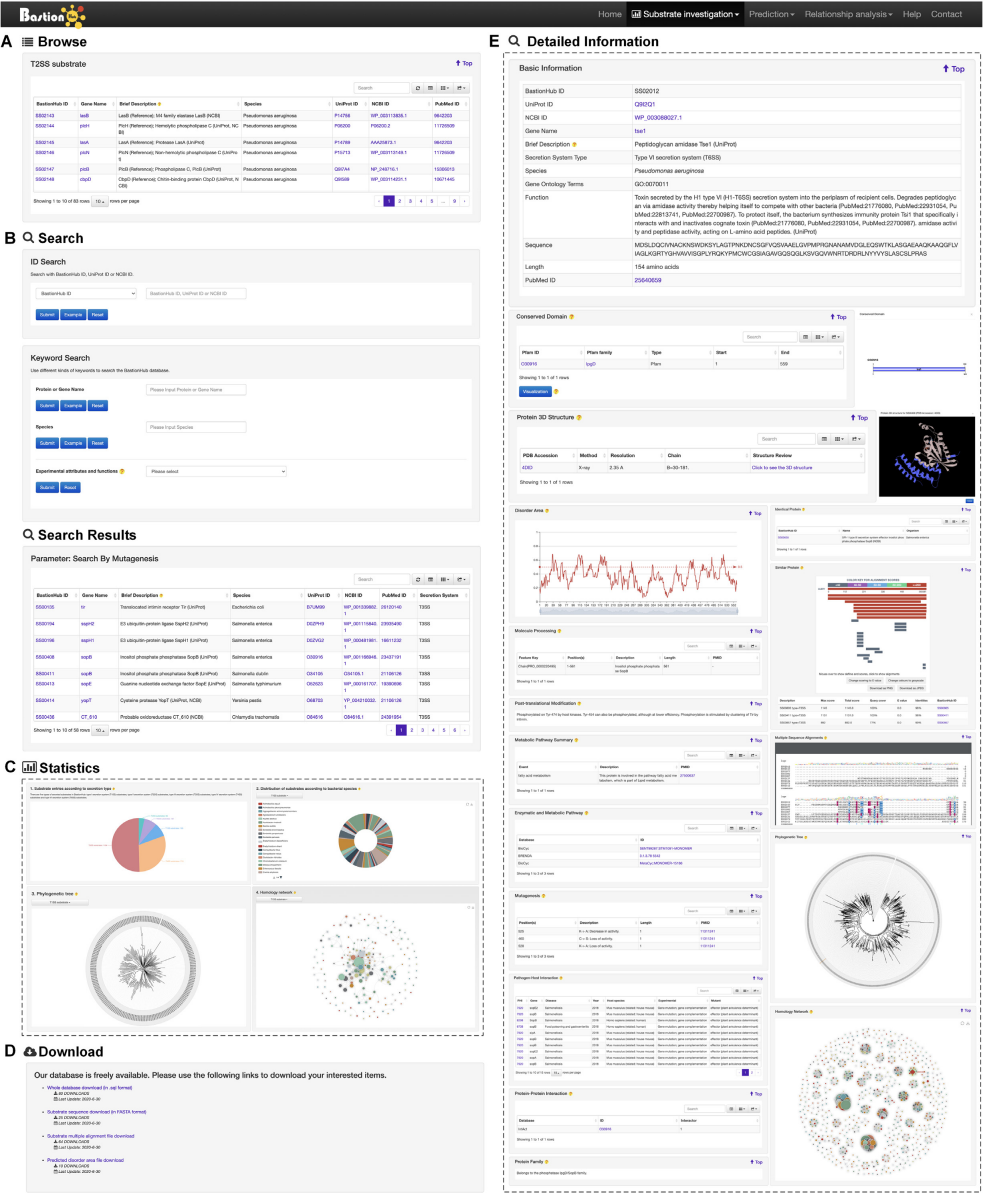
Standard investigation modules of BastionHub: the *Browse* page (**A**), the *Search* page and its results page (**B**), the *Statistics* page (**C**), the *Download* page (**D**) and the *Detailed information* page (**E**).

To allow users to explore different types of secreted substrates, BastionHub incorporates standard investigation modules including *Browse*, *Search*, *Statistics* and *Download* functions (Figures 1 and 3). These modules are located within the *Substrate investigation* tab, where each secreted substrate includes its own *Detailed information* page (Figures 1 and 3).

#### Browse

Substrates are organized by their secretion system types, and summarized with BastionHub ID, gene name, brief description, species, UniProt ID, NCBI Protein ID and PubMed ID. The display tables in the *Browse* page (and in all other pages), include sort and search functions to quickly identify substrate proteins of interest. By clicking each unique BastionHub substrate ID, users will be redirected to that substrate's *Detailed information* page for comprehensive annotations and analyses. Alternatively, users can click on the UniProt ID or NCBI Protein ID or PubMed ID to be redirected to those websites.

#### Search

This page provides users with more advanced search options than those available within the *Browse* page. The search function allows exact queries such as BastionHub, UniProt or NCBI Protein ID, or more broader queries (that do not require exact matches) using keywords, including protein or gene name and species of origin. We additionally provide a drop-down filter option to further refine results according to features such as conserved domain, protein 3D structure, molecule processing, post-translational modification, metabolic pathway summary, enzymatic and metabolic pathway, mutagenesis, pathogen-host interaction, protein-protein interaction, protein family, or identical protein. Accordingly, the *Search results* page lists the filtered substrates in a similar output format to that organized in the *Browse* page.

#### Statistics

This page contains interactive data visualization modules about the experimentally validated secreted substrates. The statistics show the distribution of substrates by their secretion types, the distribution of substrates by their species, the phylogenetic tree and the homology network for each substrate type. Clicking each section of the bar or pie charts will redirect users to a *Statistics results* page listing the filtered substrates, presented in a similar way to the *Search results* page. Clicking any substrate item in the phylogenetic tree or homology network will also redirect users to their corresponding *Detailed information* pages. Clicking any link in the homology network will display the pairwise sequence alignments between the two linked substrates.

#### Download

To assist users to work with data in batch mode, datasets and related files are available for downloading: the database in SQL format, the substrate sequences in FASTA format, the multiple alignment files, and the predicted disorder area files.

#### Detailed information

This page provides detailed annotations for each substrate comprising their basic information, advanced annotations, and relationship analyses among their associated type of known substrates. Basic information consists of their BastionHub ID, UniProt ID, NCBI Protein ID, gene name, brief description, secretion system type, species, gene ontology terms, function, sequence, length and PubMed ID. For advanced annotations, we incorporated conserved domains depicted on 2D protein maps, interactive 3D protein structures, predicted disorder area, molecule processing and post-translational modification information, metabolic pathway summaries, enzymatic and metabolic pathway details, mutagenesis results, pathogen–host interactions, protein–protein interactions and protein families. Finally, we included five pre-calculated relationship analyses for each substrate: a list of 100% identical proteins indexed by BastionHub that would normally be consolidated into a single entry, but based on their different species, annotations or sources, were kept as individual entries; a list of similar proteins within BastionHub (if available); multiple sequence alignments; a phylogenetic tree; and a homology network.

### Potential substrate prediction

**Figure 4.**
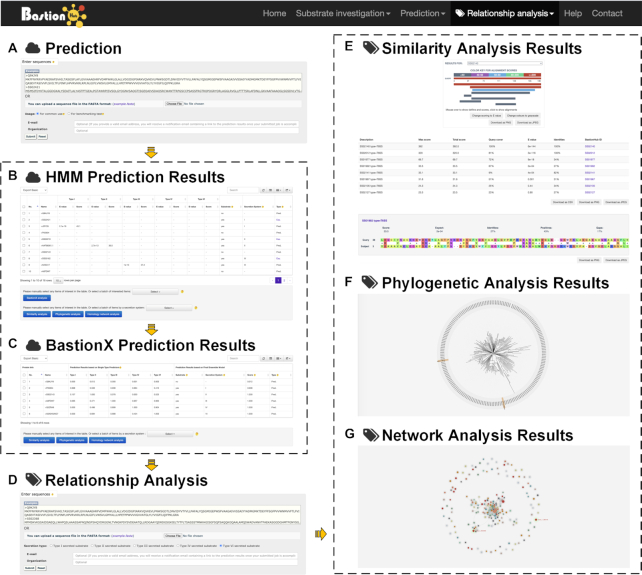
Advanced functional modules of BastionHub: the prediction input (**A**) and results pages (**B, C**), and the relationship analysis input (**D**) and results pages (**E–G**). The yellow arrows represent the interactions between modules within the BastionHub pipelines.

To allow users to predict potential secreted substrates from a list of query sequences, BastionHub incorporates two types of prediction modules within the *Prediction* tab: HMM based prediction and BastionX prediction (Figures 1 and 4).

#### HMM based prediction

We constructed a set of HMM based models using HMMER (39) to predict potential substrates for preliminary control screening. These HMM based predictors are lightweight, rapid and are ideal for even genome-scale lists of protein sequences, but will only retrieve the homologues of known substrates. Once submitted, the HMM based prediction module will provide a prediction score and E-value for each secreted protein type (if available) and select the most likely (or none) as the final prediction.

#### BastionX prediction

We further integrated the machine learning based predictor, BastionX, to achieve accurate prediction of various types of secreted substrates. Applying multiple features to learn patterns from known substrates, BastionX can be distinguished from the HMM based predictor because it can also predict novel substrates, especially those with relatively distant relationships. Once submitted, the BastionX prediction module will also list the scores for each secreted protein type and select the most likely (or none) as the final prediction.

### Relationship analyses between potential and known substrates

Considering that substrates with similar sequences may have similar structures and functions, analyzing the relationship between predicted substrates and known substrates may inspire users to infer possible structural and functional attributes that can guide experimental design. However, HMM and machine learning based models cannot be used to highlight homology relationships of potential substrates among known substrates. We therefore developed three modules within the *Relationship analysis* tab, to identify their closest homologues from known substrates (Figures 1 and 4):

#### Similarity analysis

For potential substrates, BastionHub can find their similar sequences against a user-specific dataset (i.e. type I, II, III, IV or VI substrates). In this way, one can check if a potential substrate is homologous to any known substrate. All hits to known substrates for each potential substrate will be listed and sorted according to their similarity significance. Clicking any of the known substrates will jump to its pair-wise alignments against the query protein, where the corresponding BastionHub ID link can redirect users to the *Detailed information* page for the known substrate.

#### Phylogenetic analysis

For potential substrates, BastionHub can identify their closest phylogenetic homologues against a user-selected substrate dataset. Accordingly, the relationship between potential secreted substrates and the selected set of known substrates will be depicted within a phylogenetic tree, where the query proteins are highlighted in orange, and links to the known substrates (identified using its BastionHub ID) will redirect users to their corresponding *Detailed information* pages.

#### Homology network analysis

For potential secreted substrates, BastionHub can map them onto a user-selected substrate dataset to provide a landscape of their locations amongst known substrates. This interactive network can be used to identify the closest homologues of each potential secreted substrate, where they are indicated by red diamonds. Clicking any edge in the network will show the pairwise sequence alignments between the two linked known substrates, while links to the known substrates will redirect users to their corresponding *Detailed information* pages.

### Data pipeline

Interconnecting different modules as pipelines, BastionHub can seamlessly switch between known substrate investigation and potential substrate analysis modules (Figures 1 and 4).

#### From prediction to prediction

At the HMM based prediction results page, BastionHub provides options that will allow users to feed some (or all) of the predicted potential substrates or predicted non-substrates (both marked as ‘*Pred*.’) as inputs into the BastionX prediction input page. This feature is especially ideal when using a large number of sequences to rapidly filter out homologous proteins identified by the HMM based predictor. The homologous proteins can then be further validated using BastionX; alternatively, the non-homologous proteins can be analyzed using BastionX to identify more distant evolutionary relationships.

#### From prediction to relationship analysis

At both prediction results’ pages, BastionHub provides options for users to select some (or all) of the potential substrates (marked as ‘*Pred*.’) as inputs for the three relationship analysis modules. In this way, BastionHub streamlines these naturally downstream analyses, and keeps manual selection operations to a minimum.

#### From computational results to known substrate investigation

When predicting potential substrates, BastionHub first compares them to its list of known substrates. Whenever sequences are identified as known substrates (i.e. 100% identity), these are marked as ‘*Exp*.’ in the prediction results, with links to their corresponding *Detailed information* pages. Additionally, the relationship analysis results pages also include links to dedicated *Detailed information* pages for all of the known substrates, which is especially useful for further investigation of closely related homologues to the query proteins.

## DISCUSSION

BastionHub is a universal platform developed with the intention to integrate and analyze various types of substrates secreted by Gram-negative bacteria. BastionHub provides a user-friendly, intuitive, and interconnected platform that allows analysis of known substrates, prediction of potential substrates, and relationship analysis: an all-in-one package suitable for computational and experimental biologists alike. More broadly, BastionHub showcases an extensive and interactive database, within a user-friendly framework, that could inspire more comprehensive web resource development. BastionHub will be maintained for at least 5 years and will be periodically updated to keep pace with emerging substrates and new experimental details as they become available. This will include substantial updates in the form of adding the remaining secretion system substrates, potentially including substrates from the recently proposed type 10 secretion system (44) and the Gram-positive substrates of the T7SS.

## DATA AVAILABILITY

The BastionHub platform is freely available at http://bastionhub.erc.monash.edu/. All data indexed by BastionHub can be downloaded via http://bastionhub.erc.monash.edu/download.jsp. Detailed user instructions can be accessed via the Help page at http://bastionhub.erc.monash.edu/help.jsp.
